# Positive Nosology

**Published:** 1847-01

**Authors:** 


### ART. II.

*Nosologia Positiva.* Scritta da VINCENZIO LANZA, Dottore in Medicina, Professore nella Cattedra di Medicina Pratica della Regia Universita' degli Studii di Napoli, Medico Primario e Direttore della Clinica dello Spedale della Pace, &c. &c.—*Napoli,* 1841-2.

*Positive Nosology.* By V. LANZA, M.D., Professor of the Practice of Physic in the Royal University of Naples, &c.—*Naples,* 1841-2. 2 vols. 8vo, pp. 608-760.

WE feel that we have been too long in directing the attention of our readers to these remarkable volumes; because although we have before us only two of a work which it is anticipated will extend to five, still it is of a character which, we humbly conceive, claims for the author the general encouragement of his brethren, and which promises, in return, to be of signal service to the profession, especially under the peculiar circumstances in which it is now placed. If, at the present day, there is one feature of medical literature more striking than another, it is its superfluity and redundance. With much, very much, that is excellent and valuable, there is vastly more that is trashy and contemptible. How many are the cases published which have no existence save in the distorted views of their authors? how many isolated facts brought to light with almost the assured certainty of being lost? and how many the ingenious speculations which scarcely outlive the day that gives them birth? when to these are added the many absurd professional fancies and fallacies which prevail among physicians and their patients, regarding the fashionable medical follies of the day, it must be allowed that there is here an amount of credulity and misdirected labour which with infinite benefit might be entirely suppressed. But how, it may be inquired, is this to be accomplished? We believe that, effectually, it never will;—inasmuch as the cause lies deep in human nature, and will ever and anon, in revolving cycles, be forcing itself into notice. All that can be reasonably expected is that the evil may be restrained though not suppressed; and we believe that works such as the one now before us, by enforcing better principles and especially showing a better example, are calculated efficiently to promote this most desirable end.

The range of Professor Lanza's work is most extensive, including practical medicine and medical surgery, which, scientifically considered, can never be dissociated; and its great object is to withdraw the healing art from the dominion of hypothesis "so that becoming positive" it may advance *pari passu* with the other natural sciences, and attain that elevation they are now so rapidly reaching. When, some half dozen of years ago, we had occasion to bring the subject of the science in Italy under the notice of our readers, we stated that "the Italian physic was still guided by the spirit of hypothesis;" and it would certainly be interesting if the evil in that country, long unchecked, and so rendered intolerable by its profusion, were there first to receive a check which in its influences would extend over the rest of Europe, and to Britain, which of all other kingdoms seems at present most to require the pruning of such redundancy and prurience. Nor is it the general object and end only of this comprehensive work that we would commend. The execution, as partially exhibited in the volumes before us, has been the labour of the author's lifetime, not only among his patients, but in his study; and with the most intimate knowledge of the sentiments of his predecessors and contemporaries, there is conjoined a remarkable amount of independence and originality. His views frequently startle by their boldness, as much as their novelty, and are withal founded upon a most careful scrutiny of Nature, and a most patient observance of her laws. Seeing the eminent author prominently putting forward these claims, we shall take some pains to represent the ground on which they rest; though, from the perfect originality of the views, and, we venture to add, the provincial peculiarities of his style, it is not always easily done. At the same time, we trust that our readers will not suffer from any deficiencies of ours, nor fail to derive that benefit which we believe that a patient and careful perusal of the pages themselves would assuredly afford.

The preface of a work may be considered in the light of a personal introduction, wherein the author makes his salutation and first impression on the reader. Under this conviction we give Professor Lanza's nearly entire.

"Can medicine," demands our author, "ever be rendered one of the positive sciences, more especially in its application to the cure of disease? Is it necessary ere the healing art attain a character in every way satisfactory and reasonable, that the essence of disease, and the modus operandi of morbific causes, and the appropriate remedies, be ascertained? Lastly, may the processes of ratiocination in medical science from elements which are necessarily obscure, but yet probable, be reduced to conjectures which are simple though isolated? Or, on the contrary, is it necessary, as a first principle, that we start with hypotheses more or less numerous,—hypotheses which are themselves to constitute the system of medicine? The important questions associated with these interrogatories we know, from the writings of Celsus, engaged the serious attention of the ancients, and it is high time now, as most reasonable, that, though long neglected, medical men should again revert to their most serious consideration.

"Hippocrates, as sufficiently appears from his first aphorism, was quite alive to the difficulties which must be encountered in placing the healing art among the positive sciences; but throughout his entire writings he shows that he did not consider the object unattainable; and occasionally he propounds principles in perfect keeping with the design. Celsus, in his examination of the Greek writers, clearly propounded what physic as a positive science ought to be; and accordingly, in the preface of his work, requires the Empirics to accumulate not only clear and positive medicine, but probabilities also concerning the hidden seats of diseases, their anatomico-pathological form, and the influence of their latent causes; and to reject not only from their minds, but also from the art, all that is really obscure; so that idle speculation on the essence of diseases, and the operations of causes and remedies, might be discouraged. But though this celebrated author inculcated those principles in the abstract, he did not steadily observe them in such of his writings as have reached us; nor did he proceed regularly from the lucid description of disease to its cure; but in his interesting monographs often advises remedies without assigning the reason of their selection. Hence, although we may concede to Celsus an apprehension of what physic should be as a positive science, it is certain that he left no precepts for its cultivation in this particular manner.

"Gálen was an advocate for the doctrine that the science of medicine should be based on hypotheses; commencing with disquisitions concerning the essence of disease, and the peculiar agency of causes and remedies, and that all medical reasoning should proceed from the combination of such hypotheses into systems. The influence of this celebrated physician was great, and has been felt injuriously even to the present day.

"In the progress of time physicians arose who may be designated prognosticators, or the predicating sect—*predicatori*—and who, defending experience whilst they abused systems, were useful in their way, inasmuch as they showed by facts how pure medical science, free from all hypothesis, might supply positive facts whereby the science might be enriched.

"To render medicine, however, one of the positive sciences it is not enough that old speculative systems should be condemned and abandoned; a scientific method must, moreover, be discovered, whereby, when once placed in this desirable position, it may be there retained, supplying rational principles to guide us in the art of cure. Our own age especially requires the adoption of such a method."

Our author's life, he informs us, has been devoted to this object, and he believes he can somewhat promote its advancement. A work of this kind, however, he remarks, is a task not for a single individual, but for many, and their co-operating with devotedness and perseverance. His labours, which he flatters himself will throw new light on the science, as now presented to the public, are in such a shape that every one may correct his fallacies. The critic becomes an improver, and the names of master and scholar are no longer fraught with those injurious influences which have for ages been associated with them. The wisdom of the professional chair, and the erudition of the author's pen, may hereby be readily compared with the practice of the palace, the cottage, and the hospital; and thus every cultivator of the science, in whatsoever situation he may be placed, may assist in promoting that improvement for which their united labours are so essentially required.

The FIRST BOOK of our author's work is upon THE PHILOSOPHY OF MEDICINE, a most interesting topic; and is divided into eleven chapters. It embraces discussions upon *nosology*, *natural medicine*, the errors of *transcendental medicine*, of *empirical*, *methodical*, *hypothetic*, and *eclectic*, upon the *nullity and worthlessness of the homœopathy of Hahnemann*, upon the foundation of *positive medicine*, and its *necessary criticism*. This bill of fare extends over between fifty and sixty pages; and we shall now supply a short analysis of three of the most interesting chapters. This will familiarise the reader with the distinctive characters of M. Lanza's style and manner of thought. Beyond doubt, he has most praiseworthily spent many a weary hour in studying our science and elaborating his opinions, so that many new views and incentives of thought are furnished for consideration.

CHAPTER I. The first chapter of the first book is entitled, *The necessity of a common agreement* (*consentimento*) *among physicians, in order that medicine may become one of the positive sciences.* This agreement, beyond all doubt, is a great desideratum, but is opposed by obstacles which by many will be thought insurmountable. Not so, however, in the apprehension of our author. He defines medicine to be the science which deals with the bodily infirmities of our nature; and it becomes an art when it adapts the means it possesses to assist Nature in overcoming disease. The means employed by the ancients for this important end they divided into hygienic, pharmaceutic and surgical. The word *indications* expressed the changes they wished to accomplish on the disorder; and by remedies which, according to circumstances, were either indicated or contraindicated. After the enumeration of other generalities of this kind, the author continues:

"Let us now inquire into the reason why medicine is one of the least certain of the sciences. It arises not from its own proper quality, but from the position it is artificially forced to hold. Other sciences have conventional limits for their cognizance, all exclude from their pale data that are uncertain, and rather than admit a doubt, throw the blame upon the art; and these are canons which none have thought it worth their while to dispute. Very different, however, is it with physic. Men are so anxious about their health that, rather than be sick, or run the risk thereof, they require from their medical advisers a statement of the probabilities to which they are liable, and insist upon protection and relief in the midst of doubts;—*melius remedium anceps, quam nullum.* Quadrating by these requirements, physicians are absolutely forced to admit conjectures into the science,—to deal with cases where the indications are most obscure, and adopt a treatment which is little more than tentative. In this view, physic can be little more than a conjectural science and dubious art, because what is required from it is far more than is required from any other of the sciences.

"Medicine, as a positive science, must address itself solely to the human intellect, and obtain conviction from evidence alone; it must peremptorily reject all dogmata which are incomprehensible, or beyond the grasp of the mind; whilst it retains those conjectures which will prove serviceable to the art. That individual is in ignorance regarding what he affirms who maintains, that medicine can then only be positive when it entertains nothing but what is certain. By excluding probability the indications of the art would be abandoned, and the sick would be much dissatisfied with the sacrifice. Positive medical science must, therefore, recognize the science of probabilities; such probabilities as can have a correct value attached to them, and with neither more nor less of what is their due. This condition is absolutely essential to the employment of conjecture; which, moreover, must never be employed in the support of mere hypothesis. And it must be remembered that although probabilities are thus admitted as necessary elements of medicine viewed as a positive science, still it is nothing less than the possession of what is of real and positive value, resting on the broad basis of enlarged experience and observation." (pp. 12, 13.)

The author regards hypothetical medicine in a very different light. In it the speculator fancies that he entertains the idea of a fact, which he regards as the common exponent of numerous other facts, and of the relations existing between them. Such a system is called hypothetical or systematic (an appellation which we conceive to be very unhappy):—hypothetical, because its worth is measured not by its real value, but by that which is ascribed to it; and systematic, because all the conjectures being alike supposititious, if one be erroneous, the whole fabric totters to its fall.

This clearly is the abuse, not the use of system.

"Thus," the author continues, "the different value of positive and hypothetical medicine appears as it regards the science and the art, and all who are engaged in cultivating them. The votary of medicine as a positive science, like the cultivator of the natural sciences, far from avoiding the difficulties which present themselves, investigates and overcomes them; sifting every conjecture till doubt gives place to certainty. The hypothetical physician, on the other hand, far from boldly meeting and overcoming such difficulties, avoids them; and if necessarily obliged to encounter them, endeavours so to set them aside, or smooth them down, that they shall not oppose his suppositions, or contradict his system. Positive medicine is that part of natural medicine which has been accumulated from age to age; hypothetical is artificial, has no prototype in nature, is the creation of man's fancy, the imagery of his idolatry. In a word, medicine as a positive science is a transcript of historical truth; hypothetical medicine, a romance. In positive medicine, art rules the science, since, from the prognosticated result, and the accomplishment of the expressed indication, every anticipation is realized; in hypothetical medicine, on the contrary, speculation rules art; for whatever be the issue, the practitioner has in view not so much the interests of his patient, as the protection and maintenance of the mere child of his fancy." (p. 14.)

Such is a partial analysis of the first chapter of this treatise. We proceed to the second, which more distinctly develops the author's views.

CHAPTER II. *The scientific discrimination of the facts of nosology.* In our endeavours, says the author, to create a philosophical system of medicine, and to render it one of the positive sciences, we shall attempt, first, to enumerate exactly the facts derived from clinical observation, and determine afterwards their scientific value. Disease, in his opinion, is a compound entity which, on being analysed at the bedside of the patient, may be resolved into *ten* facts, neither more nor less! Seven of these facts are possessed of a positive value, because they are directly, or indirectly, subject to the senses. They are partly cognizant to sense, partly the product of sage and indisputable conjecture. And they are the more entitled to be arranged as positive, because they may be studied as they exist in nature, and without the interposition of the slightest hypothesis. The other three facts may be designated transcendental, supposable, or hypothetical, because they are beyond the actual reach of the senses, and are merely inferences which must be drawn from supposition. Many will here be tempted to inquire if M. Lanza, notwithstanding his decided opposition to rationalism and hypothesis, has here escaped that danger he so assiduously exposes. We confess we are not prepared to give a categorical answer to this surmise. But sure we are that it is not without the most careful investigation and consideration that he has reached his conclusion. We shall notice his facts in order.

*First fact. The physiological state of the patient.* Physiological phenomena present themselves both in those in health and in those labouring under disease; and the variety they exhibit must ever command the attention of the practitioner. The physiological condition of the patient always exhibits a combination of marked appearances, which may be readily discovered upon the first examination of the patient and his disorder; and the relation between the physiological condition and the nature of the disease may usually be satisfactorily established. The conclusions are always conjectural, frequently not very evident; sometimes only probable, and occasionally dubious. The problem is to determine how much the existing and apparent irritation, increased energy, debility of the individual, and other phenomena belonging to the category, depend upon the disease; and how much, on the other hand, the disease in the various states of the patient is aggravated by these conditions. Although this fact is placed by the author in the first rank as it regards pathology, yet he states it is very generally omitted in the descriptive nosology of disease, because differing in different patients afflicted with the same disease, every practitioner regards it according to those pathological principles with which he is most familiar.

*Second fact. Combination of symptoms peculiar to disease.* All the changes which a complaint produces in the human body, whether it respects the condition of the body, the exercise of its functions, or the infliction of uneasiness and pain, are called symptoms. They are the visible elements whose union produces the external phenomena of disease; and the combination of symptoms being patent to the scrutiny of the senses, may always be examined and described. They are not all, however, of equal value in the production of disease, and should, accordingly, be distinguished as principal symptoms, and accessory ones. The former are peculiar, and so essential that they exhibit the true external characters of the disease itself. The latter do not arise directly from the disease, or necessarily appertain to it; but in whole, or in part, depend upon the peculiar concurrence of the causes, on some symptoms, as the secondary cause, or they originate accidentally. It may here be remarked that this distinction is sometimes most apparent—as conspicuous as are the symptoms themselves; whilst at other times it is made out by means of analogical conjecture. Hence the true character of the different symptoms must always be made objects of the most careful consideration.

Modern medicine has made rapid strides in discriminating the symptoms of disease, and by methods far more satisfactory than were employed by the Greeks; so that the advance of our knowledge regarding the seat of a complaint, its pathological anatomy, and the concurrence of the causes of each disease, have greatly contributed to perfect our knowledge of the exact and correct connexion of causes. At the same time it must be confessed that the moderns have not hitherto equalled the Grecian writers in the simplicity of their expositions and descriptions of disease. This may, probably, have arisen from our erroneously supposing that such pains were unnecessary; or from our being more under the influence of hypothesis than were the Greeks. So soon as medicine attains its proper position as one of the exact sciences, it will outstrip the ancients as much in this lucid and accurate description of disease as it has in the accumulation of facts.

*Third fact. Course of the disease.* The course of a disease, whether transitory or abiding, acute or chronic, continuous or periodic, stationary or declining, regular or irregular, conspicuous or latent, having remissions, alternations, paroxysms, relapses, or other variations, is sometimes so marked that it cannot be overlooked; at other times it will be readily noticed by every attentive observer; whilst in other instances it will be detected only after the most patient scrutiny. But, however this may be, there is scarcely ever room for anything like mere conjecture respecting it, and it is the occurrence of irregularities alone which demands discrimination. Upon the appearance of any such irregularity, therefore, we must ascertain its relation to the other existing circumstances, and especially to the cause of its appearance. The Greeks were particularly careful in indicating the courses and histories of diseases, and especially of acute ones; and it appears they have left us nothing to do but to follow, imitate, and equal them.

*Fourth fact. Seat of the disease.* The seat of a disease is understood to be that part, or those parts, of the body which are chiefly or solely affected by it. The external seats of disease are almost always evident, and the advance of general anatomy, physiology, and pathological anatomy is such that the seat of internal complaints may generally be readily ascertained. Hence the number of cases is inconsiderable in which the proper seat of the disease remains a matter of probability only, and still more of doubt. It is, however, true that the principal seat of a disorder is not always so apparent in internal diseases as in external ones, and especially when various and numerous parts or tissues are implicated. In cases of this kind, accordingly, the assigning the true centre and focus of the disease will always be more or less difficult and conjectural. . . . . . Modern science is far in advance of that of the ancients, as it respects the exact position of diseases; and the contrast on this point would have been still more marked, had not some modern authorities treated these physical facts so hypothetically.

*Fifth fact. Anatomico-pathological form of disease.* The visible alteration of the natural and vital properties of a part, or parts, of a patient's frame constitutes the anatomico-pathological form of a disease. Inflammation, obstruction, suppuration, may be adduced as examples. These states exhibit changes in the affected parts which are referrible to this category, and they are evident to the senses. Our acquaintance with this important subject is almost wholly due to the moderns, as the ancients were in ignorance of its very elements. It will be his painful duty, however, the author states, to explain in the next book that this fact, as marked as the preceding ones, concerning the *course* and the *seat* of the disease, has been completely obscured by the gratuitous hypothesis of some modern authors, who have endeavoured to discover one primary form of disease upon which they consider that all other morbid forms depend. If he succeeds, he continues, in demonstrating this error, and in introducing the adoption of a more judicious course, rapid strides will speedily follow in the improvement of the healing art.

The anatomico-pathological form of most external diseases is sufficiently clear, being cognizant to the senses. Some internal diseases, also, have so marked an anatomico-pathological character, that in these cases, likewise, it may be held to be decided and satisfactory. In the majority, however, of these internal disorders, it must be confessed that the character is only conjectural; and in a few instances it is still but doubtful and obscure.

*Sixth fact. Concurrence or combination of causes.* The agency of causes in producing a disease, as has already been shown, is a supposititious fact; but the combination of causes producing its effect in the production of a disease is a positive fact, and important when viewed in relation to the others with which it is here associated.

"This important fact," says the author, "has long lain latent—from the era of Grecian celebrity down to the present day; and this unfortunate obscuration has presented the chief obstacle to the advancement, not to say to the origin, of positive medicine. We were early alive to the importance of this arrestment; and by the most sedulous study for a number of years have been labouring for its removal. By a more judicious arrangement of the established classes of causes, and especially by the introduction of a new method not heretofore proposed—

namely by the prominency given to what we have termed the *primary* or *radical diseases*—we flatter ourselves that we are now in a position that we can offer this fact in connexion with those upon which we have already insisted, as a valuable addition to the science. By this distinction primary diseases are separated from secondary ones; and the true origin of all complaints may be discovered by careful investigation at the bedside of the patient, and with little fear of disappointment or error. That the combination of internal causes produces diseases is an isolated fact which is not always conspicuous, because it does not fall directly under the cognizance of the senses. But we will show that we have so far succeeded in elucidating the problem, that it may always be profitably appealed to. The particular part which each and every one of the concurrent causes plays in the production of disease will always remain conjectural; whilst, at the same time, we shall be able to distinguish betwixt those which are the true efficient causes and those which are the secondary or accessory ones. Whatever honour may accrue to us from having opened up this path, we anticipate that posterity will so improve upon it that, ere long, they will convert our probabilities into moral certainties." (p. 24.)

*Seventh fact. The agreement and tolerance of the remedial means employed.* All ingesta, whether the usual non-naturals or drugs, when swallowed by the patient, produce immediate effects, good or bad, upon the physiological condition of the patient, and more or less calculated to benefit or aggravate his pathological state. This fact, without doubt, is most important, as initiatory to all others bearing upon the cure. Accordingly, the circumstance has been conspicuously put forward by the Greeks from the time of Hippocrates, although it has not been sufficiently attended to either by them or by the more recent cultivators of the art. A neglect of this important fact has, perhaps more than any other cause, proved the rock upon which systems and improvements have made shipwreck. This criticism applies as much to the mere hypothetical speculator in physic as to the most practical of his brethren. Medicine, upon attaining the rank of a positive science, must endeavour to elucidate this circumstance above all others, as correct views concerning it will most contribute to its progress. In some instances the necessary conditions will appear conspicuous, in others doubtful, and in many obscure; and especially when, the different substances prescribed harmonizing in their effects, the favorable symptoms give place to those of an opposite kind. Willingly, says M. Lanza, will we contribute our personal knowledge, and anxiously do what we can to promote this desirable end; and it is the final goal beyond which we cannot pass.

Such, very much in his own words, is an account of Professor Lanza's seven positive facts. Next follow his three transcendental ones, which he also calls incomprehensible (*incomprensibili*), meaning thereby, we presume, not so much that they are positively incomprehensible, as that they have not yet been fully comprehended; and which may, moreover, for ever transcend the reach of man's limited powers. He thus introduces them to notice, somewhat, it must be confessed, in the *incomprehensible* style of their own nature:

"Disease, like every other entity in nature, has an essence which must consist in that change which the unknown entity displays in the course of the disease, and which is connected with the essence of vitality. The operation of the morbific cause must affect the condition of that essence of vitality necessary to constitute health, producing that special change which belongs to the particular disease it excites. Remedies, in order to cure, must operate effectually upon the essence of life, by changing the condition it has acquired in disease into that which it maintains during health. Every one must see that these three conditions are the three chief facts or circumstances, because they comprehend the intrinsic and sufficient cause of all the other facts. Hence the other seven, forming parts of the disease, must play their parts in a manner that is in conformity with the disease itself, and with the operation of its causes and remedies." (p. 26.)

But are these facts, demands the author, comprehensible? or rather, do they not transcend the powers of the human intellect? We invite not our readers to follow him in his answer to these inquiries, but proceed at once to the remaining chapter (X) of this book, on which, as before hinted, we purpose to enlarge.

*Foundation of positive medicine as a science, and an art.* 1. *As a Science.* In that system of medicine which is to be regarded as positive science, it is necessary to bring into one category all the facts that have been accumulated, such as they appear in nature; from the known and established, it must proceed to that which it does not know; and it must clearly indicate that of which it is still ignorant. It thus recognizes conjecture as a legitimate instrument and means of improvement and advance, and indicates to those who follow in its wake the path they should pursue. Hence the only object of positive medicine is to determine what is *common* and what is *peculiar* to diseases and to remedies. The science must investigate that which is common in the greatest number of diseases, and in the greatest number of cases in the same disease; and then determine what *that* is in which this community and similarity consist. It must likewise investigate what is peculiar in different diseases, and in what that peculiarity consists. This ought to be the great object of every scientific physician, and he should and will be satisfied with this grand aim in the prosecution of that science to whose cultivation he is devoted. The same remark applies to remedies. The physician should ascertain what *common* powers or virtues reside in the greatest number of remedies, and in what that virtue consists; also what is the *peculiar* virtue of each remedy, and in what that peculiarity consists; and in this matter he ought to trouble himself with no other inquiry. The science thus viewed is to be mastered by an examination, sometimes conjectural, indeed, but sound, of the seven positive phenomena or facts, as they are termed by M. Lanza, which each disease presents, as determining, not only what is *common* and *peculiar* in the disease itself, but also as ascertaining the power which experience shows every remedy exerts in the same condition of each disease. Thus, positive medicine, by cleaving to the principle of what is common and what peculiar, can alone render the curative art rational and satisfactory. The following rules are laid down for the guidance of all who would labour in this field, although, perhaps, they cannot in every case be observed to the very letter. They are altogether opposed to the mere speculator, whose one canon is, that his data and premises shall not run counter to his system:

"1st. In order to ascertain and adopt that which is *common* between two diseases, or between two cases of the same disease, and also between the powers of the remedies employed, a similarity between the observed facts must be demonstrated.

"2d. On the other hand, in order to demonstrate a peculiarity, or, what is the same thing, a difference between two diseases, or in different cases of the same disease, the direct proof arising from the examination of the different facts alone is not sufficient; it is, moreover, necessary that there be that which is common in these several items; and this demonstration must be decided and apparent.

"3d. That a perfect community and absolute peculiarity being very rare, but, in most cases, diseases and remedies offering much of what is common, with little of what is peculiar; or contrariwise, much of what is peculiar, with little of what is common, we should not in these cases unhesitatingly decide upon what is common except it were very evident, nor determine what is peculiar, unless it be equally clear." (p. 52.)

2d. *As an Art. Communia communibus, et singularia singularibus curantur.* That which is *common* in diseases being determined, not after the method of empirics, but by the sameness of the physiological state of the patient, as well as of the whole combination of symptoms; also, by the course, the seat, the anatomico-pathological form, the concurrence and combination of causes, as well as by the agreement of the remedies employed, and the patient's tolerance of them, the art must necessarily be governed by the analogy of the known common virtues of remedies. Let the proud rationalist boast as he may, he is himself obliged in practice to disregard his hypotheses; and, in analogous diseases, and in analogous cases of the same disease, he prescribes those remedies which have been found to have been useful by experience. It is clear that such analogies as are based upon the whole of the seven positive facts must prove most beneficial, whilst those of the mere empiric will for ever be despised. Induction is of the same use in the elements which are *peculiar*, as analogy is in those which are *common*. But here the question occurs, upon what rational principles can inductions be based, so as to lead to the true indication of the cure of the peculiar element in different diseases?

The errors of transcendental medicine being admitted, positive medicine must be appealed to as the only one whose principles come within the range of human intelligence. In a case belonging to the category supposed, the feelings and instincts of the patient, some fortunate occurrence, sometimes a marked trait of the disease itself, and occasionally a safe experiment or trial, supplies information regarding those curative means which the peculiarity of the disease requires. And when, as often happens, no such hint can be obtained, we must then have recourse to induction from that which is common in the given instance with other diseases, and draw our indications from analogical conjecture. Science yields no more credence to an analogy of this kind, than it draws from the groundwork of the induction; and art exerts itself with an energy proportionate to the amount of the probability. The whole history of our art exhibits the reasonableness of this principle; for it was by these means, and not by the help of mere hypotheses, that, from the very commencement, art has ever sustained itself and made progress. And throughout its onward career, as novelties and difficulties have arisen, and obstinate and unheard of cases occurred, still new methods of treatment and of cure have been discovered. For the most acute diseases hypotheses never did more; and subsequently it has fallen back again to its wonted speculation. "*Non post rationem medicinam esse inventam; sed post inventam medicinam, rationem esse quæsitam.*" From this it follows that induction in medicine as a positive science has a double object to pursue—1st, as it regards the science, it is to discover among conjectural facts the obscure elements, common and peculiar; 2d, as it regards the art, it has to discover the cure of peculiar cases, leaving the discovery of that which is common to analogy alone.

Thus, according to our plan, have we given, and without comment, a very partial and condensed epitome of Professor Lanza's first book upon the Philosophy of Medicine, insisting more particularly on the parts treating on the agreement among medical men as to the manner of cultivating their common science,—on the scientific discrimination of the facts of what our author calls nosology,—and on the foundation of positive medicine, as a science and an art. Fully are we aware that these are insufficient data, whereby any one can judge of the true value of the volumes, and it may probably exceed our powers to furnish them within our assigned limits. Nor are we disposed to follow any short or sleight-of-hand method, and say, *ex lapide Roma!* Well, however, may we here rest for a moment, and inquire what are we to think of this philosophy, and these facts? We suggest the inquiry, not so much in a spirit of captiousness as of caution. When a man for the first time turns to the elements of some even of the severer sciences, and commences with its lemmata and axioms, he may for a moment fancy that all that is in hand is sufficiently jejune and profitless, whilst in truth it is all-important. And so in the case before us. The Professor, in this portion of his work, is dealing with generalities and abstractions. His philosophy, like that prevalent among his countrymen, though much admired, is of a lofty and subtle character. It is of a species that is rare in this country, and of a class, notwithstanding all his protestations, which we cannot help arranging more with that of the speculative Idealist, of Sir James Macintosh, than with that of the Experimentalist. Our author belongs to a school very different from our own, and uses a phraseology with which we are not familiar: and who knows not that language influences thought, not less than thought language? Hence we would here tender a word of advice, to the effect, that the assiduity and enthusiasm of the Italian Professor in this early stage of its manifestation be not depreciated, and that the varied results of his painful and protracted labours be neither disparaged nor despised.

Second Book. *Clinical Pathology.* The author affixes this title to the second book, for reasons which may be best expressed by himself:

"I dare not say that I can here supply such a pathology as is peculiar to positive medicine,—such an one as it should have, and as it will ere long possess. Mere hypothetical medicine may easily invent a pathology, based upon a corresponding nosology, and emanating therefrom, because it is formed upon the theoretical principles of a system, and the treatment recommended exhibits nothing else than an artificial union of particular facts adapted to hypothetical pathological principles. The object of positive pathology, however, is very different. It has to reduce the analysis of general and compound facts, to the particulars of which they are composed, so as to determine what is *common* and *peculiar* in diseases,—in other words, the *community* and *peculiarity* of each disorder. This, again, can only be founded upon a positive nosology, because the general system can never be remembered if all the elementary facts have not been thoroughly examined. So soon as medical men concur in cultivating the science after a positive method, and our knowledge of each disease is admitted by common consent to be positive, and, as it were, perfect, then will positive pathology assume the rank it should ever maintain in our art. At present, however, and till the work here referred to be accomplished, pathology can legitimately be directed only to two objects—1st, to the exposition of what is general, in order to exhibit how very much is still unknown, and how little has yet been learned; and 2d, to indicate, by the way of clinical observation, how we are to attain, step by step, true pathological science. For such reasons as these, limiting the object of the present book, I have given it the simple title of Clinical Pathology." (pp. 57-8.)

This book contains thirteen chapters, which are mainly commentaries upon the seven positive facts already enumerated; the author still confining himself to generalities.

CHAPTER I is on *the physiological state of the patient;* by which expression is meant the vital condition in which an individual is placed when suffering under disease. We cannot, the author remarks, better estimate such a condition than by considering it in relation to the existing state of the patient, and to the energy exhibited; the whole of the functions being in this way brought under review, and life under this peculiar phasis examined. With regard to the energy—the vigour of life—we shall regard it in no other aspect than as a state of exaltation on the one hand, or of depression on the other; which conditions we avoid designating by the terms hypersthenia and asthenia, because these terms express an hypothetical cause of the condition, and the hypothesis clings most pertinaciously to the very words. In regard to this existing state, truly impossible is it that in disease the functions should maintain their natural and regular conditions; except in so far as occasionally a disorder may be of use, as well as order itself—if we be right in regarding disease as a state of disorder. In Italian nomenclature, Professor Lanza informs us that irritation is understood to be a general tremulous commotion, an agitation, in the exercise of the functions of the body. No one, perhaps, has more felicitously expressed the idea than Francesco Redi, when he said that irritation was a sedition of the vital spirits. Rejecting the hypothesis, and adopting the phrase, irritation, in the Italian acceptation of the word, may be defined to be life in a state of sedition. And thus understood, it is ever present in disease; showing itself in different degrees according to the constitution of the patient, the form of disease, and external circumstances. In mild cases, the constitution being good, the disease mild, and extrinsic excitants absent, the irritation may be in the lowest degree, and scarcely perceptible. On the other hand, whenever the disease attacks the whole frame, or even an important organ, and affects the exercise of an important function, sense, movement, or formative process, the patient never maintains his natural vigour. A change is always perceptible, and to the worse, in proportion as the disease distresses; and weakness is the invariable result. It may, indeed, happen that at the commencement of the disease the patient may appear in a state of exalted vigour; but this soon subsides, weakness supervenes, and increases in the ratio of the length and severity of the disorder. The degrees of exaltation and depression are more or less according as the constitution of the patient is more robust and hardy, or weak and feeble; also, according as the complaint entails privation or loss; also, according to the circumstances and causes in which the disorder commenced—as, for example, with copious discharges; and, most of all, according to the amount of the irritation and of pain, the worst of irritations, when intense and protracted.

Such knowledge as this is the more to be relied upon on account of its familiarity. It has been noticed from the earliest times, and is generally admitted both by professional men and others. Pathology, in its present state, can avail itself of this information only as it presents itself, and usually as somewhat generic; as is freely conceded by all. What light, then, it may be inquired, can positive pathology shed upon this information? None other, it would appear, than so far as it bears upon the physiological condition of the patient; also on the precursors of the complaint—on its course, seat, anatomico-pathological form, and its causes; likewise on the agreement and tolerance of the remedies employed; so that we may thus have general and positive rules regarding the reciprocal influence of the state of the disease, and the state of the patient. All this knowledge should be derivable from nosology, and this, conjoined to the positive method advocated above, will furnish a true pathology.

The foregoing description of increased energy, of weakness, and of physiological irritation, so frequently offers itself to observation, that it may be noticed by every one at the bedside of the patient. From the fulness of the vessels, the colour of the skin, the heat of the body, the state of the pulse, and the general debility, the inference is direct as to the excitement and depression, and every change which may have occurred; and from the symptoms—from delirium, to the most trifling alteration of function, the irritation is made manifest. To this is naturally conjoined that habit which every practitioner acquires of judging from the appearance of his patient, what previously was his state of health, and what it again would be were his trouble removed. From this the inference as to how much he is excited, and how much depressed, is at once easy and satisfactory.

*Ratio medendi. Indicatio prima sit vitalis.* In regard to the physiological powers of those agencies which operate upon the frame, they are naturally divided into revivifying (*vivificanti*), dissolving, and irritating. By the introduction of these terms the author avoids the use of the terms stimuli and counter-stimuli, which are inseparably connected with hypothetical notions, which he is solicitous should be wholly excluded from the pure matter of fact with which he wishes to deal. He defines *revivifiers* those substances which nourish the body, or which concur in doing so, increasing the means whereby it is so supported. Thus alcohol and aromatics alone would not nourish, but the revivifying powers of other substances are greatly promoted by them.

*Dissolvents* (*Scioglienti*) are those substances which being incapable of nourishing the body, allow its vitality to be exhausted, and its substance to be dissolved; and detract from the power of those substances which would nourish the body, as often as they are conjoined with them.

*Irritants* (*Irritativi*) may be distinguished into *necessary* and accidental. The necessary irritants necessarily and invariably act as such upon the whole race, upon the sound and the infirm. Frequently, however, the same substance proves not only irritant, but poisonous to one class, while it may be indifferent, nourishing, or medicinal to another. The *accidental* irritants are those substances which act on the frame, reviving or disordering, and withal disquieting and disturbing its functions, and apparently for the four following reasons: 1st. From the constitution of the patient, in reference to sex, age, temperament, habit, and especially idiosyncrasy. Thus wine is an irritant to some abstemious persons; ipecacuan, antimony, &c., to many. 2d. From the form of the disease. Thus is it with water in hydrophobia, and so with certain remedies in various diseases, or in certain states of the same disease. 3d. From the quality of the substance. And here this rule is constant, that the more powerful and energetic the article, the more likely is it to become irritative, especially when employed under unfavorable circumstances. 4th. From the time, method, or place in which the various substances happen to be administered or applied. Thus is it even with common water, if it happen, for example, to enter the larynx, or be drunk at improper times, and in unusual quantities. The application of such observations to nosology is evident, inasmuch as they are express facts, generally acknowledged and received. All that at present must necessarily be attended to is to reject them from those hypothetical uses to which the rational dynamists have applied them, and to take the high ground of adopting into our service that only which is most strikingly common in disease, and in the physiological state of the patient. Thus protesting, it behoves us to wait patiently until the progress of positive nosology has determined the relations of such physiological distinctions with the special curative powers which the articles used as remedies properly possess. Waiting in this attitude of expectancy, it appears that in the present state of clinical pathology, as it regards the regulating the powers of the patient, we can do nothing better than adopt the following maxims, which are now acted upon by all the Neapolitan physicians of standing and experience.

1st. That the physiological state of the patient be maintained in the greatest possible tranquillity, so that the powers of life may to the utmost extent be preserved, and the irritation be removed, subdued, or, at all events, not increased.

2d. That in the special cure of disease such substances as are too dissolving be avoided, so that the patient be weakened as little as possible, and the treatment may be readily tolerated. Less than this is never required in the special cure of disease.

3d. That in the special cure of disease, avoiding all agents which are too reviving, the energies of the frame be not roused more than is required; and especially that this kind of treatment do not aggravate the disease.

4th. It so rarely happens that remedies are prescribed for a disease which prove artificially irritating, whereby the treatment is hurtful, that no general rule has hitherto been provided against it, and directions must be postponed until we come to consider the treatment of individual diseases.

The foregoing is an abridged translation of the preface and first chapter of this second book, and will suffice as a specimen of the author's method of treating this part of his subject; the remainder must be given more shortly, and more in our own words.

CHAPTER II. *Combination of symptoms.* The general heads to which the symptoms of disease are referrible are two, namely, the altered quality of the body, by the moderns arranged under the head of physical signs; and the alterations in functions, arranged as physiological and pathological signs. The author informs us that it is not the object of the present part of his work to give an exposition of the external symptoms of disease, but only of such symptoms as can be demonstrated to be positive. In the present chapter the morbid symptoms are summarily announced, and in such a manner as that general conclusions may be deduced from them, and be made available to the general principles of the art.

1. *Alterations of quality* consist in physical changes of the whole body, or of some of its parts in relation to harmony, colour, heat, situation, form, density, and contiguity; and this not as a consequence of a former disease, or of original malformation, but as a necessary element of the existing disease. 2. *Alteration of function*. According to the author, sensation, vital movement, and formation are the three actions which are simultaneously maintained in the human frame, and are the manifestations of life. Every individual part, moreover, executes an office—a function which is a particular sensation, movement, or formative process, subservient to the well-being of the whole frame. Hence he retains the old distinction of functions into the animal, vital, and natural, in preference to the modern one, of those belonging to animal and organic life.

There being no disease in which the animal functions are not affected with a disagreeable sensation, with uneasiness, which again must be considered painful, such pain is to be regarded as a symptom of disease. And, as in the combination of the symptoms, as it regards the animal functions, this uneasiness and general oppression is the only common one, it may be considered as a generic sign of disease; and the severer it is the more unfavorable. If, in addition to the general uneasiness, there be also particular uneasiness, this becomes the sign of a particular disease. The disturbances of the animal functions may be divided into five classes, namely, into pain, stupor, torpor, convulsions, and aberrations. The different guises in which these symptoms manifest themselves in different diseases is reserved till the consideration of the neuronosi in general, and individual diseases in particular.

Vital movement is manifested in the unceasing current of the fluids throughout the solids of the body. The pulse and temperature, the general condition of the body, the quantity and quality of the secretions and excretions, show how they may be affected in such motions by disease. All this, however, is more particularly described by Professor Lanza, when treating of the *angionosi*, and of various individual diseases. Also, how the vital movements becoming altered, may be accelerated, retarded, or become motionless. Most diseases exhibit both an oppression of the senses, and an acceleration of the vital movements.

The formative process consists of those operations whereby the living body, incessantly composing and decomposing itself, preserves its integrity. This process continues satisfactorily only in health, and in proportion to the natural vigour of the individual. An excess of vigour produces redundance, which may be a cause of disease. Disease again always wastes the body, reducing redundancy where it exists, and subsequently preying on the frame, and leading to debility and emaciation. These several phases of the animal and vital functions is elucidated under the consideration of the *sarconosi,* and of various diseases in particular.

From this exposition it is evident that the aspect of disease in connexion with the visible alteration of functions, is thereby divided into affections of the senses, of the vital movements, and of the formative processes. In the *neuronosi,* pain, paralysis, convulsions, dementia, and such like predominate; in the *angionosi,* palpitations, hemorrhages, dropsies, fluxes, &c.; and in the *sarconosi,* cachexia, marasmus, tabes, &c. &c.

*Ratio medendi. Bona similibus, mala contrariis curantur.* In every age medicine has been searching for particular remedies for every morbid symptom; so that for a long time there has been no bad symptom without its corresponding title in the materia medica. Thus, there are narcotics, sedatives, carminatives, cordials, rubefacients, refrigerants, hemostatics, astringents, aperients, sialogogues, cholagogues, emmenagogues, expectorants, diaphoretics, diuretics, and many more. Certainly, in many instances, it is most important to relieve the urgent symptoms, because otherwise great damage may result ere the disease is removed. Hence, too, it will be seen in the nosology of such diseases, that previous to, and in conjunction with the proper cure, relief should be afforded to the dominant system. This, however, is not always easy nor possible; and especially if it be so associated with the disease, that what benefits the one is prejudicial to the other. This unfortunate coincidence must have occurred in the experience of every practitioner; and the author offers the following sage suggestions concerning it. Considering the symptom not only as in combination with the other symptoms, but also as in relation to the positive facts—to the physiological state of the patient, to the seat of the disease, its pathological anatomy, the combination of causes, and the effect of remedies, you can judge of its true nature and consequences, and determine whether it is more advisable to oppose or favour its progress. Upon this depends the proper application of the rule affixed as the motto to this chapter, *quod bona similibus,* &c.; a maxim which, though directly opposed by homœopathy, has been guaranteed by the judgment and consent of all practical observers in all ages. The symptoms being thus considered, not absolutely, but in relation to all the facts, each of which requires an indication for itself, the choice of means must be determined by a reference to them. If the immediate relief of the symptom be opposed by the physiological condition of the patient, or any other circumstance of the disease, the calculation of probabilities alone may lead to the conjecture whether any given remedy will be successful in relieving the symptoms, or be prejudicial, from being in opposition to some fact, and chiefly, perhaps, the anatomico-pathological form of the disease. Pathology in such circumstances teaches *ad quod magis urget, occurrendum;* and the urgency is not so much from the complaints of the patient, as from the danger to his life, and its being an obstacle to his cure. In such cases the choice of the remedy must be left to the experience and sagacity of the physician.

CHAPTER III. This is entitled *On the course of disease,* and must not detain us long. According to a law which the learned Professor explains in another of his works, it happens that no morbid symptom continues long in the same degree or form, but always proceeds either increasing, decreasing, or alternating, so exhibiting itself under a variety of aspects. It is by the same law that life is transitory; and disease, including its origin and termination, but a passing occurrence in life, Hence there are no truly stationary diseases, a fact which holds true even with chronic and incurable ones. Curative indications vary with the various courses which diseases present, and with the various stages which occur. Moreover, the variations which appear in such changes are not always secondary, or subordinate to the primary or principal indication which the diagnosis would lead us to expect. These changes, therefore, require to be more particularly considered under the treatment of every particular complaint. At present all that Professor Lanza attempts is to determine the primary and natural distinction of diseases, according to their course. And here two conditions are to be observed, 1st, morbid states; and 2d, morbid processes. By *morbid states* are meant those states which are maintained by an existing and present morbific cause, on which their existence immediately depends; so that when the cause ceases to operate they suddenly disappear. By *morbific processes* again, are meant such diseases as are produced indeed by a present or passing cause, but operating in such a manner that they originate a regular series of morbid events which, in regard to their course, depend no longer upon their producing causes, as they terminate only when they have run their natural and necessary course.

The *ratio medendi,* in reference to the course of disease, our author very much makes a comment upon the following axioms and phrases—*Quò natura vergit, eo ducere oportet. Dum natura movet, noli movere tu.* Every disease, he considers in its nature a combination of elements, good and bad—*bona mixta malis.* The ancients, he reminds us, were wont to refer to the various fortunes of the struggle, under the terms of *natura vincta, natura pugnante,* and *natura vincente.* Hence, according to the view which the physician takes of the part which Nature plays in each disease will be the character of his practice. If he regards Nature as always passive, he will feel urged very decidedly to interfere, and have recourse to bold sanative measures; his practice will be heroic, or, as our author has it, *furiosa.* Again, if he considers Nature to be always active in the cure, he will himself be prone to be passive, and observe the expectant plan—invariably a negligent and dilatory one. And lastly, if he look upon disease as a struggle between the complaint and Nature, he will conclude that he has nothing more to do than to restrain the vehemence of the disorder on the one hand, and to rouse Nature to the struggle on the other. In opposition to all this, positive medicine, forgetting what is passed, must commence anew the study of the powers of Nature in the cure of diseases. The facts previously expounded are the true and solid basis of such a study, the true symptoms whereby Nature shows itself vanquished, contending and triumphing, remaining still to be determined. Such an exposition as this in general terms is yet a desideratum; and in particular cases, all that it is possible to say in the present state of our knowledge will be propounded, says our author, when treating of particular diseases.

On Chapter IV, *Respecting the seat of disease,* we must also be brief. Our author views the seat of disease under two aspects.

1. An anatomical and an etiological, or as he prefers calling it, a therapeutic, point of view. The progressive advance of anatomy, physiology, and pathological anatomy makes it certain, he remarks, that no disease can be equally distributed as health is, throughout the whole body. Hence it may be laid down as a positive law, as it respects its anatomical locality, that there is no disease but what has a particular seat, and displays a particular anatomico-pathological form. At the same time, however, different diseases assume different guises, by invading, abandoning, concealing, and changing their locality. Hence various distinctions which are reserved for future consideration. At present the author only specifies such as subserve the general indications of the cure. 1st. There are diseases which have a central seat in one part only, and sometimes in one spot of this part, whilst others have their seat extended to one or more regions, or to similar parts, as to glands, muscles, serous or other tissues, capillary and other vessels, &c. &c. 2d. Some diseases remain fixed in their seat, whether central or extended, without affecting other parts, whilst others spread abroad their malign influence to neighbouring tissues, to distant parts, or over the whole body. 3d. The particular anatomical seat of a disease may be considered as necessarily elective, or changing. And 4th, there is metastasis.

2. The etiological or therapeutic seat differs widely from the anatomical; because wherever situated it produces a general effect, and is hence a morbific cause. Diseases, therefore, may certainly be distinguished into general and local, in reference to their respective causes. Hence those are called general which, however much they may be concentrated anatomically in one part, or extended into tissues, and remain there, or may shift about, yet art, for their removal, must employ general means; in other words, remedies whose powers extend over the whole body. Local diseases again are such, that whatever may be their anatomical seat, experience shows that every attempt at what may be called a general or constitutional cure is useless, and that they are to be removed only by dislodging them from their favorite haunt. By this arrangement and definition, Professor Lanza considers we get rid of all questions concerning the inscrutable essence of morbific causes, and their inscrutable operations upon life; since by changing the etiological question into a therapeutic one, we confide the decision to experience. Accordingly, then, as experience, from such rational principles, indicates the diseases wherein a general or a local cure may be the more suitable, we are led to expect it in the particular treatment of a disease.

CHAPTER V. *On the anatomico-pathological form of disease.* We find the interest of the work increase with acquaintance, and the author's views appear more important as they are developed. This fifth chapter is perhaps the most important we have yet encountered, and we cannot therefore pass it slightly by. Professor Lanza regards pathology as a branch of nosology, a union which has not been generally recognized; and hence the departments have been severally cultivated as if they had no alliance with each other. He endeavours to supply a general view of pathological anatomy, pure, positive, and absolutely free from hypothesis, whereby it has heretofore been so greatly injured. He divides morbid appearances into nine forms; including alterations, lesions, degenerations, pseudo-organic productions, fitozoid productions, sarconotic entozoi, organic vices, morbid habitudes, and dissolutions; under this last are comprehended gangrene, sphacelus, and death. In thus treating the subject he believes he is rendering pathological anatomy a service, by demonstrating the true and positive method in which it ought to be cultivated, and ensures to nosology, whilst treating of each disease, the possession of the fifth positive fact, constituting the pure science of the anatomico-pathological form of every disease. To these we must shortly advert.

1st. *Alteration.* The author gives this name to those morbid forms in which there is a simple change of structure in a part, which does not require any change of anatomical condition to reduce it again to the sound state; so that if lesions, degenerations, &c., of any kind be added, these evidently originate in the alteration, necessarily preceding, accompanying, or following, but not as necessary or integral parts of it. Alterations usually originate of themselves, and are simple and alone.

*Inflammation, obstruction,* and *congestion* are next in a similar way defined; and it is then noted that the pathological *community* of these morbid forms is evident, whilst the *peculiarity* will be insisted on under the particular diseases. These three forms may have their seat in any texture of the body, and they run into each other. Inflammation may be acute, or rapidly chronic; obstruction may be chronic, or slowly acute. Congestion may appear in either of these forms, and any of the three may modify the others. The same internal morbific cause may, under varying circumstances, produce any of the forms. Lastly, there is nothing more important in practical therapeutic analogy than what is exhibited in such morbid states, respecting the agreement and tolerance of the remedies which may have been employed. Sometimes two or more of these morbid forms appear combined together, a state which has been called *sub-inflammation.* To this term the Professor has no objection; because it cannot be used as if implying that the state specified has any control over the other conditions, or more, at all events, than they have over it. It is nosology which must ascertain the peculiarity of each state. To this section the author adds the general indications of cure, as he does to the following. Here we must refer to the work itself.

*Neuronosi.* The neuronosi, or diseases of the nerves, are the next variety of affections which are introduced under this head. Though not so well defined as the preceding by visible changes in the anatomical condition of the parts, yet the physiological symptoms, or the alternation of the function, as pain, stupor, convulsion, &c., are most marked. Not that these express the nature of the affection. Hence the neuronosi, as diseases of the nerves generally, compel us to have recourse to anatomico-pathological investigation. This department of our science arranges under two heads the anatomico-pathological forms assumed in the diseases of the nerves. *Common* neuronosi are those which consist of any pathological form, as inflammation, congestion, obstructions of all lesions, degenerations, pseudo-organic productions, sarconotic entozoi, organic vices, and dissolutions attacking the brain, medulla spinalis, as organs, in the same manner as they attack other parts of the body. *Peculiar* neuronosi again, consist of those in which there is a morbid alteration of the intimate structure of the proper substance of the brain, medulla spinalis, and nerves. It is apparent that such neuronosi have no claim at present to hold a place in pathological anatomy, because we are quite ignorant of the true nature of the alteration. They ought, nevertheless, to have a provisional not a hypothetical place, especially in reference to nosology.

*Angionosi.* The angionosi include such diseases of the blood-vessels as are not represented by anatomico-pathological characters, but only by the combination of symptoms peculiar to the functions of the parts. Among these are abnormal movements of the heart and arteries, morbid states of the temperature, as heat and cold, hemorrhages, dropsies, and also all excesses, deficiencies, and deviations of the excretion and discharge of the secondary fluids. It is clear that the angionosi do not form a primary morbid form, but one that is always secondary, or, in other words, dependent upon some alteration, lesion, degeneration, or other morbid form ; and that the symptoms are referrible chiefly to the functions of the vessels, and manifest themselves not so much from a particular state, as from peculiar circumstances which must be indicated by nosology. The author here postpones to a future time the hemorrhages, and other forms of these affections, because the peculiarities they present will then be studied practically, so that the angionosi like the neuronosi may have in pathological anatomy a general ascertained principle in the commonality and peculiarity which nosology discovers in practice.

The author's general views of *fever* must not be passed over ; but whether they refer to continued fevers in their varied forms, or to the fever which accompanies inflammation, does not clearly appear ; they probably, however, include both. He observes that there may be inflammation so slight, and so confined within the affected part, that it does not excite marked fever. Boerhaave taught this ages ago, and re-echoed herein the sentiments of Hippocrates. Hence, if there is a primitive form of any disease, be it obstruction, neuronosis, lesion, or other, and if fever be added, it is held, and with the concurrence of physicians of all ages, that inflammation will be found participating in the constitution of the anatomico-pathological forms of the disease, either as preceding, succeeding, or accompanying, and from the character and vehemence of the fever, the share which the inflammation contributes to the disorder may be determined. Modern pathological anatomy, with great advantage to practice, has elucidated a proposition which is the reverse of the foregoing, namely that not only does inflammation cause fever, but that there is no fever which is not dependent upon or represented by inflammation. The ancients did not state this, because they were ignorant of the extended seats of diseases, and of the spreading of inflammation into tissues, as exhibited both in the living and the dead. Fever having an extensive range, the ancients held it to be a primary morbid form, independent upon any known inflammation ; and hence they called it essential or idiopathic. The speculations of the Rationalists on the point have hitherto been most vague and unsatisfactory, and it has been an observation of our own day only, that fever is always a symptom, and of all symptoms the most certain, of the vehemence and character of inflammation, whether its seat be confined to one part, or extended into a tissue.

2d. *Lesions.* Wounds, contusions. Here we must be short, though the author is most minute and ample in his details, in some points impugning some of the most popular doctrines of the day, we allude to some of the most important doctrines of inflammation, and likewise anticipating opinions which have been put forth with high claims, and we humbly conceive, not more high than just, to novelty.

These lesions are divided into—1st, *Concussion;* 2d, *Ecchymosis;* 3d, *Ecchymoma,* soft tumours from circumscribed ecchymosis; 4th, *Contused Wounds;* 5th, *Compound Wounds.*

These morbid forms may present the combination of their symptoms under three pathological phases. As *lesions,* they induce symptoms of wounded anatomical integrity of the part, and corresponding to the physiological exercise of its functions. As *irritants,* they produce a morbid state represented by symptoms of disturbed vitality in the injured part. As *morbid processes,* they invariably produce inflammation when the divided parts are not immediately healed, and the effused and extravasated fluids speedily absorbed.

*Ratio medendi.* Healing or incarnation is the natural reunion of parts which have been divided. The ancients believed incarnation to be a purely physiological fact, entirely a work of Nature, and one of the manifestations of the natural formative power of every living body. John Hunter, however, conceived that for the effecting of this work, Nature must participate in the diseased process; and, more particularly, that a slight inflammation must be lit up in the wound, an inflammation to which he gave the name of adhesive, and which he conceived to be essential for every reparatory process. On the wide-spread prevalence, we might say the universality of this opinion we need not enlarge. But surely, says our author, this Hunterian notion of adhesive inflammation is nothing better than a capricious fancy, and one which is opposed to the first elements of our physiological knowledge of the formative process in living bodies. As early as the year 1825, Professor Lanza opposed this hypothesis, and demonstrated then, as he is prepared to do again, that not only the natural carnification, but also that morbid adhesions, as they are called, are not to be ascribed to disease under any modification, but are entirely Nature's work. So too it happens, that when during the healing of a wound there is slight inflammation or phlogosis, this adjunct does not prove an obstacle to the cure, any more than an assistant to the natural powers, in the healing process. Here we cannot forbear inquiring, if this be aught else than the doctrine of Macartney, and an anticipation of Dr. J. H. Bennett's *normal nutrition?*—his statement that the exudation of blood plasma is the essential phenomenon of inflammation; and that this process is a modification of the function of nutrition as explained by the doctrine of cytogenesis? We have not hitherto been able to ascertain the fact, whether the opinion of the Neapolitan physician is based upon the same careful inquiry into the results of modern researches, made by means of organic chemistry and the microscope, as has been that of our acute and intelligent countryman. If so, all the more creditable. But be this as it may, the coincidence appears remarkable, and cannot fail to be satisfactory to the distinguished teachers whose views on this important matter are so far coincident.

The ratio medendi succeeds; and long disquisitions concerning suppuration, the varieties of pus, sanies, &c., and other matters more particularly belonging to medical surgery, which we must pass by.

3d. *Degenerations.* This section must be summarily passed over. It comprehends the subjects of *hypertrophy*, and *atrophy*, relating to structure; and of *induration* and *softening*, relating to the consistence of the part.—So, too, with

4th. *Pseudo-organic productions*, which the author arranges according to their appearance, under six divisions, namely, *morbid adhesions, false membranes, transformations, excrescences, anomalous tumours*, and *anomalous substances*, under which last title are considered *melanosis, colloid* matter, *cyrrhosis, silerosis*, and a fifth, to which he has given the name of leuconosis.

5th. *Fitozoid productions*. Concerning this important group of disorders, our author states that he considers himself even now entitled to give a positive definition, inasmuch as he is convinced that it has an accuracy which will speedily be universally conceded to it. He defines fitozoid productions, those bodies which have an inherent life and peculiar structure, which take origin in the living frame, infect it, increase at its expense, and there remain implanted as fitozoi, or parasitic animal plants.

He divides them into two genera: 1st, those which are essentially malignant, having a special tendency to degenerate, to consume the part in which they are located, and exhaust the constitution. Such are *scirrhus, fungus hæmatodes, encephaloid*, and *tubercles*. The second genus consists of such as are not essentially malignant, not having the same spontaneous tendency to fatal degeneration. At the same time they may acquire this tendency from disease, bad treatment, or other causes; such are *polypi, lypomi, sarcoma*, and *cystic* growths. The peculiar attributes of these productions the author states will be more fully expounded in the book on sarconosi, and under those diseases to which they have the nearest nosological arrangement. In this place he gives only their general anatomico-pathological characters,—for which, we need not add, we have no room.

6th. *Sarconotic entozoa*. Among the vermes, or rather those tiny creatures which are engendered and lodged within the human frame, there are some which merit consideration less from their natural-history characters, than from their vitiating the parts in which they may be lodged. In this way they produce diseases which are generally arranged with sarconosi; and hence M. Lanza designates them *entozoi sarconotici*.

Of this number are *hydatids*, which, as now demonstrated, are endowed with vital properties, and live at the expense of the structure in which they reside. Anatomically they appear like clear vesicles filled with fluid, or with lymph of various composition, as will be subsequently noticed particularly. Meantime be it remarked that they possess all the characters of fitozoid productions, raised, however, to a higher state of existence, so that they have a more decided inherent life, and are less dependent upon the parts among which they lodge; so that they are more in mere contact with them, that implanted in the locality where they originated. Hence pathological anatomy rigorously maintaining its pretensions to a positive science classes hydatids as entozoi. For nosological reasons, however, our author deems it improper to separate them from the helmintosi; he, therefore, confers upon them the epithet of sarconosi, places them among the sarconotic diseases, and there assigns them their place among the anatomico-pathological forms.

Among swine there are examples of the death of such entozoi, of their dissolution, and the absorption of their cysts, and the final cure of the disease. No such occurrence has yet been witnessed or accomplished in man. Hence it remains for the art, by the help of analogy, to obtain their eradicative cure; and, in the meanwhile, endeavour to alleviate the symptoms, and oppose their injurious effects.

7th. *Morbid habits of body.* By a morbid habit of the body is understood that combination of symptoms which produces a general effect upon the whole frame, and is characterised by the general diseased appearance of the whole man. The following states belonging to this category are brought under review: *turgescence, languor, cachexia, tabes, marasmus, caccochimia,*—a good habit of body to appearance, with signs of the operation of particular or general morbific causes.

These are never primary but always secondary affections. But concerning them we must be silent.

8th. *Organic vices* succeed, which may be either acquired or original; and may be divided with a reference to their effects as being—1st, merely *deformative,* interfering with the natural appearance without producing any other annoyance; 2d, as *morbiferent,* acting as causes producing or aggravating disease; and 3d, as *mortiferous,* by impeding the exercise of some important function, and so producing death. As to the nature of these affections it may be considered as they influence the size, harmony, continuity, form, colour, appearance, and structure of the part, which is done accordingly, at length in the volume.

Chapter VI. The sixth chapter is entitled *the concurrence of causes in producing diseases;* and here we have room for little more than cataloguing the causes enumerated by the author, displaying in striking terms how minutely and comprehensively he has gone to work.

I. *Producing causes,* according to the part they play in inducing disease, may be classed as follows: *disposing causes, occasional, efficient, accessory,* and *concurrent;* in all five.

II. *Natural causes* may be arranged under seven heads: 1st, the *natural anatomical structure of the parts,* as of the brain in apoplexy, and the lungs in phthisis; 2d, *peculiar physiological idiosyncrasy,* in reference to the peculiar mode in which a morbific agent or a remedy may act in certain persons; 3d, *age;* 4th, *sex;* 5th, *temperament;* 6th, *hereditary predisposition;* and 7th, *habitude,* by which nature is much influenced—a second nature. The first six of these can be only disposing causes; the last, besides being disposing, may also be preparatory, and, in other cases, occasional; because it may render a man liable to disease to which he was not naturally disposed, or may slowly prepare him for an attack to which he was not otherwise inclined. The efficient cause, it should be remarked, is often found among those regarded as constitutional or habitual, because it receives its character from the habits of the patient; and, in fact, has no other cause than his habitual neglect and disregard of the rules of health. The power of art is naturally limited in these constitutional disorders.

III. *Non-natural causes* are then enumerated; and

IV. *Unnatural causes,* so called, because, by their peculiar influence, they predominate over the natural and non-natural causes. Four of these are enumerated *epidemics, endemics, contagions,* and *poisons.*

V. *Radical causes.* Some diseases possess two constant etiological characters; the former, that they never originate as effects or transmutations from other diseases, each having its own proper cause, natural, non-natural, unnatural, and often unknown; the latter, that they always act as causes—disposing, occasional, efficient, concurrent, &c. From this it follows that, etiologically, diseases may be divided into two classes; in other words they originate, on the one hand, as radical primary diseases; or, on the other, originate as secondary, resulting from the foregoing, and following them in their origin, condition, and issue. These radical diseases of M. Lanza amount in number to twenty-two, and may be divided into three categories: 1st, *vitiating radical diseases*; 2d, *excreting radical diseases*; and 3d, *virulent radical diseases.* In the first category are included, 1, habitual catarrh; 2, colonosi, bilious disorders; 3, habitual plethora; 4, idioneuronosi, nervous diseases; 5, helmintosi, worms; 6, hemorrhoids; 7, uric lithiasis, gravelly complaints; 8, gout; 9, the sequelæ of periodic disorders. In the second category are arranged the primary excretory diseases: thus, 10, herpes; 11, scaldhead; 12, chilblains; 13, the sequelæ of acute contagious diseases, and 14 suppressed secretion of milk. The third category consists of the virulent radical diseases, and contains, 15, tinea; 16, rickets; 17, scrofula; 18, scurvy; 19, syphilis; 20, scabies; 21, leprosy; 22, sarconotic tendency.

The author conceives that the greatest service he has rendered to positive medicine, is that he has created an etiological science for these twenty-two radical diseases. He maintains, moreover, that unless due attention be paid to them, the curative art can never render the principles of the indications which are directed against the causes certain.

VI. *Injuring causes.* Finally, we have to add that injuring causes are introduced, and are divided into external and internal. The *external* injure mechanically and chemically. The *internal* are impurities, morbid products, &c., which generate within the body, injure the parts where they collect, and vitiate it either mechanically or chemically. Among the mechanical effects are to be placed all those mechanical lesions which were enumerated in the preceding chapter, (the fifth) and considered as causes of the secondary diseases originating from the mechanical disturbance, distension, and pressure they occasion. The chemical, again, consist in the materials retained, collected, stagnant, absorbed from one quarter, and elsewhere deposited. Such are—1st, blood, inclosed in aneurisms, collected in ecchymosis, and effused in cavities, so as to be removed from the influence of vitality; 2d, the secondary fluids when they become stagnant in their canals, and especially if they have been previously altered by serious disease,—as vitiated bile, dysenteric fluid, and, beyond all others, urine when long retained; 3d, matters absolutely morbid, as the fluid of dropsy, pus collected in abscesses, or effused, as in empyema, the sanies of cancer, and all other degenerating sores, &c. &c.; 4th, worms, wherever they make their appearance, indurated fæces, &c. &c.

The curative art, in treating external causes belonging to this section, must adapt particular means to particular cases. In internal causes a primary indication is to oppose by every means the morbid product, and, if possible, to remove the morbific cause; a palliative indication is to alleviate the symptoms, and the secondary effects produced by these offending causes.

CHAPTER VII, *On the tolerance and conference of the remedies employed,* must, for the sake of brevity, be omitted, and we must draw our analysis to a close by a short account of the succeeding one.

CHAPTER VIII. *The distribution of diseases, into classes,* or, in other words, the *nosological arrangement* of our author. Two questions are first of all put. First, whether the nosologies of Sauvages, Sagaro, Pinel, Vogel, Cullen, Frank, and many more, afford a natural arrangement? This is answered in the negative, And secondly, whether they are practically useful? and here the finding is, that, though studied by scientific men, they are disregarded by practical ones.

Professor Lanza submits the results of his labours as a small contribution towards the natural distribution of diseases; and first separates the twenty-two radical complaints, which we have previously had occasion to enumerate, from the others which are more or less to be regarded as secondary. In establishing these secondary diseases, having no wish to pervert Nature, or interpret her doings where indications are insufficient, he has always preferred and followed what is useful, and associated those diseases which have so much of what is *common* in them, that the art actually assigns to them an analogous treatment and cure.

FIRST CLASS. *Radical diseases.* In this class is included diseases differing in appearances, course, form, and cause; but agreeing in this, that each has a peculiar and primary cause which may be the root of every other disease, and present an obstacle to every mode of cure; on this account they are named radical, as controlling the curative art.

THE SECOND CLASS consists of *acute inflammatory diseases.* These diseases have a common course and anatomico-pathological form, consisting of inflammation concentrated in some part. They are made to precede rather than follow fevers, because they throw much light on the diagnosis of these latter.

THIRD CLASS. The *acute continued fevers* have also a common course. Their anatomico-pathological form, as revealed by pathological anatomy, consists in inflammation, which may pervade any of the tissues of the frame.

FOURTH CLASS. *Periodic endemic fevers.* These exhibit such a singularity in their course, and owe their origin to a cause so peculiar, that however much they may resemble the continued fevers in their anatomico-pathological characters, yet requiring particular curative indications, they have been placed in a distinct class.

FIFTH CLASS. *Transitory contagious diseases* have an acute course, and an inflammatory anatomico-pathological form, quite agreeing with that of inflammatory diseases and continued fevers. The whole of them have not their seat in the skin, and hence the name of exanthemata is not bestowed upon them. Other complaints having an analogous cause are brought under the same head. The cause of them all is a contagion peculiar to each, but *common* in respect of its transitory effect, which commonness throws much light upon their treatment.

SIXTH CLASS. The *sarconosi.* Under this class the subjects both of growth and wastings are included; nor can these, henceforward, ever with propriety be dissociated. How could fitozoid productions be handed over to the surgeon, whilst tubercles, so essential a part of the history of phthisis, be retained in medical nosology. That which is common in the appearance, course, form, and cause of these complaints is so marked that they must occupy a single class.

SEVENTH CLASS. The *angionosi* comprehend those diseases which exhibit, as a marked symptom, functional disorder of the heart and blood-vessels. Hence the class includes the anatomico-pathological affections of the heart and blood-vessels, hemorrhages, dropsies, effusions, and morbid collections. The present state of the science requires that these diseases should be united in the same way as if they actually had been so.

EIGHTH CLASS. That of *neuronosi* comprehends those diseases which exhibit well-marked symptoms of altered function of the nerves. It is of importance to distinguish these with the degree of accuracy that is necessary to guide us in the art of curing them.

NINTH CLASS. *Diseases peculiar to women.* This class comprehends those complaints which, appearing to have their efficient causes in the sex, are natural to them. Among these are hysteria, chlorosis, diseases connected with the catamenia, the puerperal state, suckling, &c. The ordinary complaints which attack women in common with the other sex, and sometimes preferably are, of course, excluded.

TENTH CLASS. *Diseases peculiar to children.* This class includes those diseases only which have their efficient and natural cause in the age of the patient. All complaints, therefore, are to be excluded which attack them in common with adults, and sometimes it may be in preference. The ratio medendi is by this arrangement much elucidated, and great advantage, as in the case of female complaints, accrues from considering that which is common in their respective categories.

This constitutes the whole of Professor Lanza's nosology; and under this arrangement he treats of all the diseases to which man is heir. Very different is it from any previously proposed, and on its real merits it is not easy to decide until the details are reviewed and scrutinized.

The six remaining chapters of this book treat, in a general way, of the *distinction of remedies,* the *diagnosis* of disease, the *prognosis,* the *cure generally,* the *methods by which the cure is to be promoted,* and lastly, the *method in which positive medicine is to be advanced.* With a few sentences upon this last important topic we must bring the survey of the whole chapter to a close.

According to the Professor, the chief obstacles which impede the advance of experimental medicine are the three following: First, the extreme variety which prevails in different countries, provinces, and cities, in the municipal customs, plans of cure, popular remedies, and medical usages, generally, whereby all common grounds of comparison are wanting. Secondly, there is the monster abuse of polypharmacy, on which nothing need be said, the injury it has caused alike to humanity and the art being notorious. Thirdly, there is the number of compound remedies still in vogue, a clear relic of barbarism, which should long, ere this time, have been banished from amongst us. Leaving all this behind, positive medicine must commence anew to determine the true physiological and pathological power of remedies, and in order to do so must proceed in the following manner:

1st. We must ascertain the real effects of remedies on the healthy frame. Such experiments, however, must not be executed after the fashion of the rationalists, or of M. Hahnemann, by employing the most powerful and extraordinary remedies, and in doses which prove irritating, and almost poisonous. On the contrary, we must employ remedies of medium power, common, ordinary, and perfectly well known; and we must adapt their doses to the display of their true normal effect, whence we shall be able to appreciate their virtue and efficiency on the physiological state of the patient.

2d. Having determined the physiological power of ordinary remedies, we must then use them in the most simple manner, and solely in the cure of those common diseases with whose nature we are best acquainted. It is thus alone that we can become familiar with the physiological and pathological power of a number of common remedies in common diseases.

3d. Step by step, from the knowledge of the ordinary powers of common remedies, we must subsequently proceed to that of the more powerful remedies in rarer diseases; holding it clear that our advance will be more sure and certain, in proportion as such a mode of experimenting becomes more common and familiar.

We have no doubt, adds the enthusiastic Professor, that medicine will have such a progress; and, in order the more rapidly to advance it, he should like to see the arduous labour undertaken by a society of enlightened men collected in one of the great capitals of Europe which, a centre of scientific intercourse, had correspondents throughout the world. The united efforts of such a union might succeed in greatly expediting the removal of the three great obstacles to the advance of the healing art upon which we have previously dwelt, not less than promote its progress by accomplishing the three most desirable objects we have just enumerated.

Thus have we presented our readers with an analysis of the earlier portion of our author's volumes, full as our space admits, and the manifest merits of the work demand. It includes the whole of the author's exposition of his general doctrine, which must have cost him an amount of careful consideration to which, we doubt not, he attaches a high value. But although the most original and novel part of the work, it does not, therefore, follow it will prove the most popular or pleasing; on the contrary, it may be quite the reverse. The dealing with general principles and abstractions, however imperatively necessary and important, suits not every taste. Our object, however, has not been so much merely to please, as within a moderate space to convey distinct ideas of the peculiar character of this portion of his labours, the exposition of which, we trust, will prove both interesting and useful.

The concluding part of the first volume and the whole of the second are occupied, as the remaining ones will be, with the application of the principles above propounded to the details of disease under its multifarious forms, and the best method of encountering and, if possible, relieving and removing it. The third book, accordingly, is occupied with the twenty-two radical diseases, already catalogued, in so many distinct chapters; the fourth, with common continued fevers; the fifth, with periodic endemic fevers, and the sixth with acute inflammatory diseases, whose consideration, if at all, must be reserved for a future opportunity.

Meanwhile we feel entitled neither to ask the judgment of others, nor to volunteer our own—definite and conclusive—as to the merits of this work. We have been much struck with its originality, with its rich store of facts, and the industry and succcss with which they have been grouped and generalized. We have also been surprised to find that the whole plan is so ingenious, pregnant with thought, and endowed with good sense and sound discretion. We have throughout, ourselves been anxious to follow an advice preferred by M. Lanza, which we recommend to all: "Let the reader examine with a kindly spirit what we have proposed as the best method of studying disease in a positive way; let every one endeavour to find a better,—we doubt not there are many,—*e viva felice!*"

---

